# Stability Dynamics of Plant-Based Extracellular Vesicles Drug Delivery

**DOI:** 10.3390/jox15020055

**Published:** 2025-04-13

**Authors:** Satyavati Rawat, Sanchit Arora, Madhukiran R. Dhondale, Mansi Khadilkar, Sanjeev Kumar, Ashish Kumar Agrawal

**Affiliations:** 1 Department of Botany, Kurukshetra University, Kurukshetra 136119, Haryana, India; satyavatirawat1988@gmail.com; 2 Department of Pharmaceutical Engineering & Technology, Indian Institute of Technology, Banaras Hindu University, Varanasi 221005, Uttar Pradesh, India; sanchitarora.rs.phe24@itbhu.ac.in (S.A.); madhukirandr.rs.phe22@itbhu.ac.in (M.R.D.); mansi.khadilkar.phe22@itbhu.ac.in (M.K.); 3 Department of Dravyaguna, Faculty of Ayurveda, Institute of Medical Sciences, Banaras Hindu University, Varanasi 221005, Uttar Pradesh, India; kumarsanjeevdg@bhu.ac.in

**Keywords:** plant-based extracellular vesicles, stability, exosomes, storage, therapeutic

## Abstract

Plant-based extracellular vesicles (PBEVs) have been recognized for their wide range of applications in drug delivery however, the extent of their medicinal applicability depends on how well they are preserved and stored. Assessing their physicochemical properties, such as size, particle concentration, shape, and the activity of their cargo, forms the foundation for determining their stability during storage. Moreover, the evaluation of PBEVs is essential to ensure both safety and efficacy, which are critical for advancing their clinical development. Maintaining the biological activity of EVs during storage is a challenging task, similar to the preservation of cells and other cell-derived products like proteins. However, despite limited studies, it is expected that storing drug-loaded EVs may present fewer challenges compared to cell-based therapies, although some limitations are inevitable. This article provides a comprehensive overview of current knowledge on PBEVs preservation and storage methods, particularly focusing on their role as drug carriers. PBEVs hold promise as potential candidates for oral drug administration due to their effective intestinal absorption and ability to withstand both basic and acidic environments. However, maintaining their preservation and stability during storage is critical. Moreover, this review centers on the isolation, characterization, and storage of PBEVs, exploring the potential advantages they offer. Furthermore, it highlights key areas that require further research to overcome existing challenges and enhance the development of effective preservation and storage methods for therapeutic EVs.

## 1. Introduction

**Figure 1:**
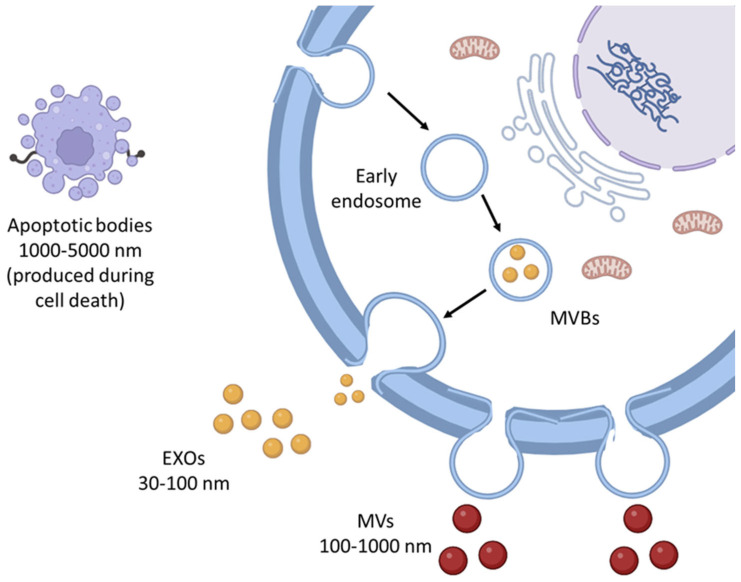
Major classes of EVs include EXOs, MVs, and apoptotic bodies, each with distinct biogenesis pathways and functional roles. EXOs are typically 30–100 nm in diameter and originate from the endosomal pathway, while MVs (also known as ectosomes) are larger, ranging from 100–1000 nm, formed through direct budding from the plasma membrane. Apoptotic bodies, the largest type of EVs (1000–5000 nm), are released during cell apoptosis and contain cellular debris and organelles.

Extracellular vesicles (EVs) are vesicles expelled by cells into the extracellular environment and play a key role in intercellular communication. They are broadly classified into three categories: apoptotic bodies, microvesicles (MVs), and exosomes (EXOs), each with distinct production pathways. Apoptotic bodies, the largest EVs with a size range of 1000–5000 nm, are produced during cell death, and MVs, which range in size from approximately 100 to 1000 nm, are formed through the direct budding of the plasma or cell membrane. In contrast, EXOs are smaller, measuring between 30 to 100 nm [1,2]. These membrane-bound nanovesicles are generated when endosomes bud inward, leading to the formation of multivesicular bodies (MVBs). These MVBs then fuse with the cell membrane, releasing multiple EXOs simultaneously (Figure 1). Both vesicles facilitate intercellular communication by sorting and carrying cargo information, such as DNA and RNA [3].

Due to their nanoscale size, EXOs are capable of passive targeting of tumors through the enhanced permeation and retention (EPR) effect. This property makes EXOs particularly effective in delivering chemotherapy drugs, offering enhanced efficacy and reducing side effects. Tian et al. [4] demonstrated that EVs could be used to deliver integrin-targeted, dendritic cell-derived chemotherapies into mice with two different tumor models, achieving superior outcomes compared to the administration of the free drug. Similarly, Tang et al. showed that paclitaxel-loaded EVs were more effective in inhibiting metastases of Lewis lung cancer than the standard formulation of the drug, Taxol. These studies also revealed that EV-based treatments could overcome common side effects associated with free drugs, such as liver or kidney dysfunction. Although these studies establish the therapeutic potential of EVs, there remains a gap in understanding how different factors-such as EV source, stability, and scalability affect their clinical translation. Addressing these aspects is essential to remove beyond experimental findings toward practical medical applications. In another study by Tang et al., intraperitoneal (i.p.) injection of EVs loaded with cisplatin significantly improved long-term survival in mice with ovarian cancer-bearing tumors. Following these promising results, phase II clinical trials have been initiated to evaluate the efficacy of EV-encapsulated chemotherapy drugs in treating malignancies associated with ascites and pleural effusions in patients with advanced cancer. In these trials, patients are administered the treatment locally four times weekly, with both therapeutic outcomes and adverse reactions carefully documented [5].

Despite the clinical potential of EVs, the sources of EVs significantly impact their feasibility as drug carriers. Human cell derived EVs pose challenges related to batch-to-batch variability, high production costs, and regulatory concerns regarding safety [6]. In contrast, PBEVs offer a sustainable, cost-effective alternative with comparable physicochemical properties and potential therapeutic application. While previous reviews have extensively covered various aspects of PBEVs, to the best of our knowledge, there is currently no dedicated review article specifically addressing the stability of PBEVs. This review fills this gap by providing a comprehensive analysis of the stability challenges associated with PBEVs, incorporating discussions on emerging characterization techniques, formulation strategies, and innovative stabilization approaches that have not been extensively covered in existing literature. Moreover, our review aims to offer a unique perspective by integrating recent advancements in the field and proposing strategies for improving the long-term stability and scalability of PBEVs for therapeutic applications.

In addition to their role as drug transporters, various sources of EVs have been explored, with milk-derive EVs showing particular promise. Milk-derived EVs have been reported as effective carriers and delivery vehicles for miRNA, siRNA, protein cargo, and other biomolecules [7]. Remarkably, the physical characteristics of EVs derived from cow milk remain stable even after four weeks of storage at −80 °C, with no significant change in the percentage of paclitaxel retained within these EVs. Moreover, milk EVs stored at −80 °C for several months exhibit minimal loss of activity and largely remain free from coagulation [8]. However, variability in EV stability has been observed in different sources. For example, studies indicate that while cow milk EVs remain stable under low temperatures, raw human breast milk stored at −80 °C or 4 °C can undergo degradation, leading to loss of cellular viability. Such discrepancies highlight the need for systematic stability studies across various EV sources to optimize their storage and therapeutic potential.

EVs derived from human cells face numerous challenges, particularly concerning scalability in manufacturing and high costs, especially when isolated for drug delivery purposes. In contrast, EVs derived from plants are emerging as promising alternatives. Several studies have shown that plants secrete EVs with morphological characteristics similar to those released by eukaryotic cells. Transmission electron microscopy (TEM) has revealed that PBEVs possess a spherical shape with a bilayer membrane and an electron-dense core, closely resembling human EVs [9]. The presence of vesicles similar to EXOs released from the MVBs of plant cultures was first reported by Halperin and Jensen [10]. Subsequent studies confirmed that PBEVs originate from MVBs that fuse with the cell plasma membrane, akin to the process observed in eukaryotes [11]. The endosomal sorting complex required for transport (ESCRT) machinery, which includes ESCRT I, II, and III but not ESCRT-0, along with accessory proteins, plays a critical role in the MVBs-dependent formation of EVs in both plants and eukaryotes [12]. To compensate for the absence of ESCRT-0 in plants, several candidate molecules have been proposed, including the FYVE domain protein, which is essential for endosomal sorting, and the orthologue of mammalian TOM-1 [10]. Recent comparative studies further suggested that the biogenesis mechanisms of plant and mammalian EVs are not only structurally similar but also functionally analogous, particularly in their ability to transport bioactive cargo for intercellular communication [13].

While the mechanisms underlying EV secretion in plants and mammals share similarities, unique features of PBEVs remain largely unexplored. Exocyst-positive organelle (EXPO)-mediated secretion, autophagosome-mediated secretion, and vacuole–PM fusion have also been described as alternative pathways for biogenesis apart from the multivesicular bodies-dependent PBEV secretion [14,15]. Despite these advancements, the mechanism by which EVs cross the cell wall remains unclear. It has been suggested that certain hydrolases associated with EVs and their lipidic structure may facilitate their passage through the cell wall pores [16,17]. Several studies on PBEV structure and cargo have revealed their potential physiological role in responding to pathogens, interacting with microbes, and organizing cell walls [18,19,20]. This underscores the significance of plant EVs in plant defense and cellular organization. In the realm of clinical research, one trial is exploring the ability of PBEVs to deliver curcumin to colon tumors. The trial measures outcomes such as curcumin concentrations in normal and cancerous tissue after oral administration, as well as the safety and tolerability of this approach compared to curcumin alone, as determined by the occurrence of adverse events [21]. Although such trials demonstrate the feasibility of PBEVs in drug delivery, systematic stability studies are needed to ensure reproducibility across different plant sources, isolation methods, and storage conditions.

PBEVs present several stability advantages over other types of EVs. As an extractive natural product, PBEVs offer numerous benefits compared to synthesized nanoparticles. Notably, they are non-cytotoxic, and their natural membrane envelope facilitates easier absorption by target cells while protecting nucleic acids from environmental stressors and enzymatic degradation. Edible plants, in particular, are a rich natural source of EVs, enabling large-scale extraction with high yields. Additionally, EVs derived from these plants are non-toxic and non-immunogenic, making them especially suitable for administering medications and nucleic acids [9]. Despite their potential, systematic stability studies on PBEVs are still lacking, and this review aims to address this gap by evaluating their stability in comparison to conventional EVs.

Recent studies have shown that EVs are present in food, and upon consumption, they interact with human metabolism in significant ways. These EVs carry various bioactive compounds that can modulate multiple metabolic pathways after being absorbed in the intestines. For instance, EVs derived from edible plants have been shown to stimulate the expression of genes related to anti-inflammatory cytokines and antioxidant compounds, thereby helping to maintain intestinal homeostasis [9]. What makes PBEVs particularly interesting is their ability to naturally enhance human health without causing side effects. They possess therapeutic properties, including anticancer, anti-inflammatory, and wound healing activities. The membranes of PBEVs have a distinctive lipid composition that provides them with exceptional resistance to chemical and physical stressors. This resilience makes them ideal candidates for engineering and drug loading, offering great potential in targeted delivery applications [22]. Multiple studies on eukaryotic EVs have shown that the nucleic acids they contain are resistant to degradation by body fluid enzymes. This property has been leveraged to promote the use of PBEVs as an effective nucleic acid delivery method. For instance, research has demonstrated that grapefruit-derived EVs loaded with miR-17 successfully inhibited the growth of brain tumors in mice [23]. Similarly, ginger-derived EVs loaded with siRNA proved effective in treating ulcerative colitis [24]. Advances in engineering techniques have enabled the incorporation of various nucleic acids, including short RNAs, mRNA, and exogenous DNA plasmids, into PBEVs [9]. This capability suggests that PBEVs could serve as versatile carriers for nucleic acids of diverse sizes, broadening their potential applications in therapeutic interventions.

Altogether, EVs hold significant potential to impact therapeutic targets in various ways. They are often considered desirable for therapeutic development due to their ability to mediate beneficial effects, such as tissue regeneration [25], antigen presentation [26], and antimicrobial activity [27]. However, it is crucial to recognize that EVs can also have adverse effects, including promoting tumor vascularization [28], inducing stress reactions with bystander effects [29], and contributing to inflammatory and autoimmune conditions [30]. Therefore, it is essential to fully understand the potential pathogenic consequences of EVs before advancing to human trials and market release. Despite these challenges, EVs are valuable as biomarkers because they can be detected in blood and urine for a wide range of diseases, including cancer and cardiovascular disorders [31]. Their high biocompatibility and inherent targeting capabilities also make them promising candidates for drug delivery [31]. However, the long-term stability of PBEVs, particularly under different storage conditions, has not been well characterized in previous reviews. This review seeks to address this gap by evaluating PBEVs stability in comparison to conventional EVs. This review delves into the sources and isolation methods of PBEVs, emphasizing their importance in therapeutic drug delivery. By critically analyzing the factors influencing EV stability-including biogenesis, storage conditions, and processing methods-we aim to provide a comprehensive understanding of their potential for therapeutic applications.

## 2. Sources and Isolation Techniques for PBEVs

**Figure 2:**
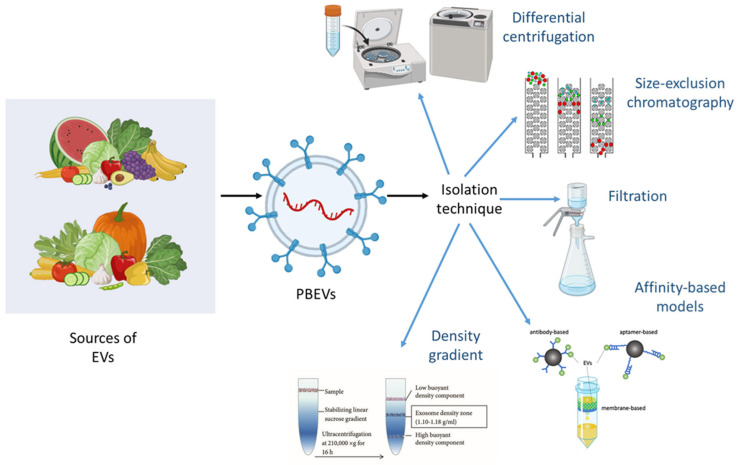
Extraction and isolation techniques for PBEVs include several key methods. Differential centrifugation involves multiple spins at varying speeds to separate PBEVs based on size and density. Additional methods such as size-exclusion chromatography, filtration, affinity-based models, and density gradient centrifugation enhance the purity and yield of PBEVs by effectively separating them from other cellular components and containments. Each technique contributes to optimizing the extraction process for functional and characterization studies of PBEVs.

The isolation of EVs remains a significant challenge despite decades of research and the development of advanced methodologies. It is well-known that separating EVs from various sources requires specific and stringent techniques to ensure purity and functionality. PBEVs, however, offer a more accessible alternative. As a natural product, they can be refined using simple extraction methods, bypassing the need for expensive and complex cell culture conditions. Additionally, PBEVs are well-suited for drug delivery applications because they can carry and transmit a large number of biomolecules [9,22,23,24]. The choice of isolation technique is crucial as it influences subsequent analytical processes, and the limitations of each method may introduce biases in the outcomes. Several techniques are commonly employed for EXO isolation, including differential centrifugation [32], density gradient centrifugation, size exclusion chromatography, affinity-based methods, and filtration (Figure 2). Each method has its advantages and drawbacks, and the selection of an appropriate technique depends on the specific requirements of the research or application.

### 2.1. Differential Centrifugation-Based Isolation

Differential centrifugation, a technique where the acceleration of the centrifuge determines the size of the particles being separated, is one of the most widely used methods for isolating EXO like EVs [33] including PBEVs [34]. The process begins with modest centrifugation speeds to remove residual cells. Larger vesicles, such as ectosomes, are typically isolated at higher accelerations (around 15,000–18,000× *g*), while the smallest vesicles, such as EXOs, require ultracentrifugation at much higher speeds (approximately 100,000–120,000× *g*). However, the standard techniques for isolating EXOs using differential centrifugation can sometimes lead to challenges. For instance, particle aggregation may occur in highly concentrated solutions [35], and freezing EXOs can cause structural damage, leading to irregular biological activity. Another significant drawback is the co-precipitation of protein aggregates, apoptotic bodies, and cell debris during the separation process. To address this, additional washing and centrifugation steps are often recommended to remove protein clumps and other contaminants.

Beyond differential centrifugation, other methods for PBEV isolation are being explored. While these techniques are still in the developmental stages, they show promise for improving the purity and functionality of isolated PBEVs. One example is the use of the interactions between phosphatidylserine (PS) and T-cell immunoglobulin mucin-4 protein (Tim4). This method is based on a Ca^2+^ dependent binding reaction, where EVs can be detached by adding calcium-chelating agents, offering a potentially more selective and efficient approach to PBEV isolation [36].

### 2.2. Density Gradient Separation

The density gradient method is a refined technique utilized to separate vesicles based on their size and density. In this process, vesicles are placed within a gradient medium, allowing them to migrate to the point where their density matches that of the surrounding medium. This method is particularly effective for isolating EVs with densities ranging from 1.13 to 1.19 g/cm^3^. To establish the necessary density gradients, substances like sucrose are commonly employed. Additionally, various commercially available kits are used, including Nycodenz, Accudenz, Histodenz (all based on iohexol), and OptiPrep (based on iodixanol). Despite its effectiveness, one of the challenges associated with the density gradient method is the unintended co-isolation of high-density lipoproteins (HDL) within some gradient media, which can complicate the purity of the isolated EXO preparations [37].

### 2.3. Size Exclusion Chromatography for EV Purification

This approach is analogous to column chromatography, but it separates vesicles based on their size. The process begins with the elution of larger particles from the column, as these particles are too large to enter the pores of the stationary phase. In contrast, smaller particles get trapped within the pores and remain in the filtration column for a longer duration. Porous beads and Sepharose CL-2B are commonly used as the stationary phase in this method. Typically, a centrifugation step precedes the filtration process to remove cell debris and other large contaminants, ensuring that only the desired vesicles are subjected to size-based separation. This method is effective in refining the purity of isolated vesicles by leveraging the size-selective nature of the stationary phase [38].

### 2.4. Affinity-Based Isolation Methods

This method is widely used for isolating PBEVs by leveraging specific proteins present on their surface. The process involves using appropriate macromolecules, such as antibodies, peptides, or aptamers, to selectively isolate PBEVs. However, the method’s effectiveness is limited by its selectivity, as it may exclude some desired PBEVs that do not display the necessary surface antigens for binding to the selected antibodies. One common approach within this method is magnetic bead separation, which relies on immune affinity. In this technique, magnetic beads coated with specific antibodies capture the targeted PBEVs [39]. After incubation, the beads with the bound PBEVs are removed from the medium using magnetic force. This technique’s performance can be further enhanced when combined with various analytical tools, such as miRNA isolation kits or ELISA tests (e.g., ExoTEST™).

To increase the yield of PBEVs isolation, additional precipitation agents can be employed. In some studies, PBEVs were centrifuged at low acceleration in the presence of polymeric precipitation buffers, often provided in commercially available kits like ExoQuick or its modifications. However, these methods are generally more effective for isolating smaller vesicles (60–180 nm) and are not typically suitable for ectosome isolation. Additionally, the co-precipitation of protein aggregates has been observed, which can complicate the identification of surface antigens in various analytical methods, such as flow cytometry [40]. This limitation underscores the ongoing need for refinement in these isolation techniques to improve the specificity and purity of the isolated PBEVs.

### 2.5. Membrane Filtration Approaches

Unlike the other isolation methods that focus on extracting particles from a sample, filtration works by concentrating vesicles dispersed in a large volume of liquid. During this process, excess fluids, such as conditioned cell culture medium or body fluids like urine, are discarded. Filtration can also serve as an additional step in the isolation and purification of EVs. In this method, the sample is manually pressed through membrane filters. Particles smaller than the filter’s pore size pass through, while larger particles are trapped and can be recovered later. One of the advantages of this method is that the pore diameter can be adjusted to isolate particles of different sizes, and the efficiency of filtration-based isolation is comparable to that of ultracentrifugation [41]. However, uneven recovery of different proteins from the membrane remains a challenge. This issue can be mitigated by using alternative vesicle recovery methods or membranes with lower protein affinity.

To address potential vesicle deformation, gravitational or low-pressure filtration followed by an additional purification step is recommended. Dialysis membranes, including hydrostatic filtration dialysis (HFD), have also been applied in PBEVs isolation. For instance, Musante et al. recently described using HFD to isolate EVs from urine samples [41]. This method involves filtering a sample through a dialysis membrane with a molecular weight cut-off (MWCO) of 1000 kDa, preceded by centrifugation at 2000× *g* to remove cell debris, bacteria, and free proteins. Sodium citrate and EDTA are added to prevent calcium oxalate crystallization. After filtration, samples can be further centrifuged to separate EXOs and ectosomes. HFD has been shown to yield good results in subsequent protein and RNA analyses, such as mass spectrometry and Western blotting [41]. Despite these advantages, the time-consuming nature of such procedures can potentially affect sample composition.

### 2.6. Advanced Isolation Techniques for PBEVs

The isolation of EVs from plant sources presents unique challenges due to the presence of cellular debris, proteins, and polysaccharides that can interfere with purity and yield. Traditional isolation methods such as differential centrifugation, density gradient centrifugation, size exclusion chromatography, affinity-based separation, and filtration have been widely used, but they come with limitations related to throughput, specificity, and vesicle integrity. To overcome these challenges, recent advancements in microfluidics, acoustofluidics, deterministic lateral displacement (DLD) have been explored for PBEVs isolation, offering enhanced efficiency and reproducibility.

#### 2.6.1. Microfluidic-Based Isolation

Microfluidics-based EV isolation has gained attention as a promising alternative due to its ability to process small sample volumes with high precision [42]. By utilizing microchannels and tailored flow patterns, microfluidic devices can separate EVs based on size, charge, or surface markers, eliminating the need for extensive ultracentrifugation. Dielectrophoretic separation within microfluidic systems applies an electric field to sort vesicles based on their dielectric properties, while immunoaffinity-based microfluidic platforms selectively capture PBEVs using antibodies against specific surface proteins [43,44]. Additionally, viscoelastic flow separation enables the continuous and label-free sorting of EVs in viscoelastic fluids, improving sample purity. These approaches are particularly valuable for PBEVs, which often require minimal processing steps to maintain bioactivity.

#### 2.6.2. Acoustofluidics-Assisted Separation

Acoustofluidics, an advanced technique combining acoustic waves and fluid flow dynamics, has also emerged as a non-invasive method for PBEV isolation. This approach leverages surface acoustic waves (SAW) and bulk acoustic waves (BAW) to manipulate EVs based on size and density, allowing for precise separation without chemical modifications [43]. Acoustofluidics is especially beneficial for isolating EVs from complex extracts, as it preserved vesicle integrity and minimizes aggregation. The ability of acoustofluidics systems to operate in a continuous-flow manner enhances scalability [45], making them suitable for large-scale production of PBEVs for biomedical and therapeutic applications.

#### 2.6.3. Deterministic Lateral Displacement (DLD) for EV Sorting

Another promising approach is DLD, a microfluidic technique that enables size-based EV separation by directing particles through an array of micropillars within a microchannel [46]. This method provides high-resolution separation, eliminating the need for multiple centrifugation steps while maintaining EV integrity [43]. DLD has shown significant potential in fractioning PBEVs from background contaminants [47], offering a reproducible and efficient alternative for research applications. Recent research on PBEVs extraction and isolation is summarized in Table 1.

Based on a thorough analysis of the literature, differential centrifugation remains the most commonly used technique due to its accessibility and cost-effectiveness. However, it presents limitations related to contamination with non-vesicular components. Density gradient centrifugation offers higher purity but is time-consuming. Size exclusion chromatography has emerged as a promising alternative, particularly for applications requiring high yield and purity. Affinity-based methods provide high specificity but may result in selective loss of vesicles. Filtration, while effective in concentrating vesicles, is often used as a supplementary step. Overall, for PBEVs, size exclusion chromatography appears to be one of the most efficient techniques, balancing purity, yield, and reproducibility.

Recent advancements in microfluidics, acoustofluidics, and DLD have significantly improved the efficiency and reproducibility of PBEV isolation. Among these, microfluidics-based isolation shows great promise due to its ability to process small sample volumes with high precision. Acoustofluidics, which use sound waves for label-free separation, preserve vesicle integrity and allow for continuous flow separation, making it suitable for large-scale applications [48]. DLD provides high-resolution size-based separation without the need for ultracentrifugation [43]. When comparing these advanced methods, microfluidics appears to be the most versatile and scalable approach, whereas acoustofluidics offers a non-invasive alternative that retains vesicle bioactivity.

**Table 1:** Summary of extraction/isolation method employed for PBEVs.

S. No.	Method of Extraction/Isolation and Purification	Plant Source	Disease/Application	Reference
1.	Differential centrifugation and sucrose gradient ultracentrifugation	Grape-fruit	DSS-Induced colitis	[49]
2.	Centrifugation and ultracentrifugation	Panax Ginseng	Cancer immunotherapy	[50]
3.	Centrifugation and column filtration	Broccoli	Colitis	[51]
4.	Ultracentrifugation	Apple	GI tract diseases	[52]
5.	Differential Centrifugation	Strawberry	Oxidative stress in human mesenchymal stromal cells	[53]
6.	Ultracentrifugation and tangential flow filtration	Aloe vera	Wound healing	[54]
7.	Ultracentrifugation, electrophoresis combined with dialysis	Lemon	Gastric Cancer	[55]
8.	Differential centrifugation and ultracentrifugation	Celery	Tumors	[56]
9.	Ultracentrifugation and sucrose gradient centrifugation	Turmeric	Murine colitis	[57]
10.	Differential centrifugation	Blueberry	Immunomodulatory Therapy	[58]
11.	Ultracentrifugation	Ginger	Inflammatory bowel disease	[59]
12.	Ultracentrifugation and sucrose gradient centrifugation	Garlic	Obesity-induced systemic and brain inflammatory activity	[60]
13.	Centrifugation and Ultracentrifugation	Black Bean	Cancer Therapy	[61]
14.	Centrifugation and sucrose gradient ultracentrifugation	Tea flower	Breast Cancer	[62]
15.	Differential Ultracentrifugation	*Catharanthus roseus*	Anti-tumor, Anti-helmintic, anti-diabetes	[63]
16.	Differential Centrifugation	Asparagus cochinchinenis	Hepatocellular carcinoma	[64]
17.	Centrifugation and ultracentrifugation	*Lonicera japonica*	Intraepithelial neoplasia caused by Human Papillomavirus	[65]
18.	Centrifugation	*Centella Asiatica*	Anti-proliferative activity	[66]
19.	Centrifugation, ultracentrifugation, size exclusion chromatography	Sesame	Anti-inflammatory	[67]
20.	Size exclusion chromatography and ultracentrifugation	Cabbage and Red cabbage	Inflammation and inhibition of apoptosis	[68]
21.	Size exclusion chromatography and High-pressure homogenization (HPH)	Cucumber	Improved dermal penetration	[69]
22.	PEG-based precipitation	Ginger and grapefruit	COVID-19	[70]
23.	Cold maceration technique	*Terminalia chebula*	Hepatocellular carcinoma	[71]

## 3. Therapeutic Applications of PBEVS in Drug Delivery

EVs isolated from various fruits and vegetables contain several active constituents that are beneficial in treating various conditions. Their intrinsic biocompatibility, stability, and ability to traverse biological barriers make them superior candidates for natural drug carriers. This capability allows EVs to be directly utilized for disease treatment. Numerous studies have supported the possibility of complexing EVs with antioxidants such as vitamin C, catalase, and superoxide dismutase. Unlike synthetic nanoparticles, which often suffer from rapid clearance and systemic toxicity, PBEVs provide a natural lipid bilayer that protects encapsulated antioxidants from enzymatic degradation and oxidative damage, thereby enhancing their bioavailability [72].

A notable example of harnessing the potential of PBEVs is presented by Raimo et al., who developed a method to isolate EXOs from various fruits and vegetables and then concentrate these EXOs in the presence of fruit juices to encapsulate the antioxidants within them. This technology, called Exocomplex^®^, is patented and was designed to help recover from H_2_O_2_-mediated damage and restore redox balance. The innovation of Exomplex^®^ highlights the scalability and commercial potential of PBEVs in nutraceutical and pharmaceutical applications, where natural antioxidant therapies are gaining increasing traction. The preparation of EXOs in this manner aimed to improve the stability and bioavailability of antioxidants. Common fruits like asparagus, kiwi, cherry, lemon, mango, papaya, and grapefruit were used to extract juices. The resulting EXOs contained antioxidants such as catalase, glutathione, superoxide dismutase, ascorbic acid, ATP, and other phenolic compounds [73]. However, one of the major concerns in utilizing PBEVs for clinical applications is the risk of pesticide residue, heavy metal contamination, and microbial presence. Thus, rigorous purification methods such as ultracentrifugation, tangential flow filtration, and chromatography should be explored to ensure their safety and efficacy.

In addition to this passive encapsulation method, several active loading methods exist for introducing cargo into EVs [74]. Active loading approaches, such as sonication, extraction, and electroporation, enhance drug loading efficiency but may also compromise vesicle integrity. Most of these methods involve manipulating EV structures to load exogenous substances, which can potentially alter the stability of the vesicles compared to those that are naturally bioactive. For instance, studies suggest that excessive shear forces from sonication and extrusion could disrupt EV lipid bilayers, leading to leakage of encapsulated cargo [75]. To date, there are no published studies on the storage stability of exogenously loaded EVs and their cargo. However, evidence suggests that the loading mechanism can impact EV properties. For example, electroporation is a commonly used method for loading cargo into EVs for gene and drug delivery. This process temporarily forms pores in the EV membranes, allowing cargo to diffuse into the vesicles. Although widely used, electroporation has been shown to induce nucleic acid precipitation, such as siRNA, and result in low siRNA incorporation into EVs [76]. This limitation has led researchers to explore alternative techniques, such as chemical transfection or saponin-mediated loading, which offer better cargo retention while minimizing vesicle damage. While the endogenous functionality of the EVs in vitro may remain unchanged, Johnsen et al. reported that electroporation can lead to EV aggregation [77]. The same study also suggested that RNA might be released from EVs or degraded after electroporation depending on the buffer used, but further research is needed to confirm this and clarify the dynamic nature of any cargo loss or degradation. These findings emphasize the urgent need for comparative studies evaluating different loading techniques to identify optimal conditions that balance cargo loading efficiency and EV integrity. Additional studies are also required to characterize the effects of other EV loading methods on potency and morphology after storage.

The therapeutic potential of EVs for delivering exogenous pharmaceutical agents has been demonstrated in several studies. The first such instance was in 2010 when Sun et al. showed that EVs derived from EL4 cells and complexed with curcumin exhibited improved circulation and targeting compared to the free drug [78]. This pioneering work laid the foundation for using EVs as bioinspired drug carriers, prompting subsequent research into PBEVs as efficient delivery vehicles. Since then, various research studies have been conducted, with active ingredients being loaded into EXOs for therapeutic delivery applications. For instance, Tian et al. recently engineered EXOs to express iRGD peptides, enhancing doxorubicin delivery in vivo. A direct comparison of PBEVs with mammalian-derived EVs for doxorubicin delivery could reveal key differences in uptake efficiency, immune evasion, and systemic clearance. In cases where obtaining sufficient quantities of EVs is challenging, EXO-mimetic vesicles have been utilized for doxorubicin delivery [79]. However, a major challenge in using PBEVs is standardizing their isolation and purification processes, as batch-to-batch variations could impact their therapeutic consistency. Thus, regulatory frameworks must be established to ensure the reproducibility and safety of these natural carriers. Table 2 provides various examples of therapeutic applications of PBEVs.

## 4. Stability Considerations and Characterization of PBEVs

The stability of PBEVs is one of the most critical factors determining their utility as therapeutic drug delivery vehicles. Stability issues affect the preservation of their structure, surface characteristics, cargo integrity, and biological activity during storage and transport, all of which can influence their effectiveness as drug carriers.

Compared to EVs derived from animal and bacterial sources, PBEVs exhibit unique stability characteristics due to their distinct lipid composition and the presence of plant-derived bioactive compounds. EVs from animal sources are typically rich in phospholipids and cholesterol, which contribute to their membrane rigidity and stability under physiological conditions [96]. In contrast, PBEVs often contain plant sterols and flavonoids, which can enhance their resistance to oxidative stress but may also alter their structural integrity under different storage conditions [97]. Similarly, bacterial EVs known as outer membrane vesicles (OMVs), possess a robust peptidoglycan layer that confers high structural stability, making them more resistant to pH fluctuations and enzymatic degradation compared to PBEVs.

### 4.1. Key Factors Affecting the Stability of PBEVs

#### 4.1.1. Impact of Environmental Conditions

A crucial environmental factor impacting PBEV stability is temperature. PBEVs are relatively stable at ultra-low temperatures, such as −80 °C or in liquid nitrogen, where their structural and functional integrity can be preserved for extended periods. Kim et al., [98] explored the stability of Dendropanax Morbifera leaf-derived PBEVs under various storage temperatures (−20 °C, 4 °C, 25 °C, and 45 °C). After purification, the PBEVs were diluted in distilled water and stored at these temperatures. Over 4 weeks, pH remained stable at −20 °C and 4 °C but slightly declined at higher temperatures. Vesicle size increased over time at all temperatures, with −20 °C proving most effective in maintaining size within 30–500 nm. Protein content also decreased across all conditions. Freeze-thaw cycles (−20 °C to room temperature) led to vesicle aggregation. A similar study on Kaempferia parviflora rhizome vesicles showed that −20 °C and −80 °C conditions preserved vesicle properties like shape, size, and surface charge more effectively than 4 °C and room temperature. Repeated freeze-thaw cycles caused size enlargement and decreased surface potential, reinforcing the importance of stable, low-temperature storage for vesicle integrity [99].

The membrane of PBEVs is sensitive to pH changes, which can affect their structure and drug-carrying ability. PBEVs are generally stable at neutral pH (6.8–7.4); however, extreme pH conditions lead to rapid degradation. In acidic conditions (pH < 4), the membrane integrity of vesicles is compromised, leading to cargo leakage and vesicle rupture. Initially, the pH of leaf-derived EVs (LEVs)-TMO had a pH of approximately 5. Over the 4 weeks, the pH of LEVs and LEVs-1,3-BG showed a slight decrease, with the total change being less than 1 unit. In contrast, the pH of LEVs-TMO remained stable at around 5 throughout the experiment, across all TMO significantly minimized the pH variation of LEVs stored under different temperatures for 4 weeks [98]. In addition to temperature and pH, oxidative stress can also adversely affect PBEV stability. Exposure to oxygen can trigger lipid peroxidation in the vesicular membrane, especially in vesicles rich in unsaturated lipids [100].

Compared to EVs from other sources as animal and bacteria-derived EVs, PBEVs exhibit unique stability characteristics. Animal-derived EVs are often more sensitive to enzymatic degradation due to their high lipid and protein content, requiring strict storage conditions to prevent rapid breakdown [101]. Bacterial EVs, on the other hand, possess more rigid membrane structures due to the presence of peptidoglycan and lipopolysaccharides, providing enhanced stability under extreme conditions such as high temperatures and low pH [102]. However, PBEVs have been reported to show higher stability in a broader range of pH values (neutral to mildly acidic) compared to mammalian EVs, making them advantageous for oral and topical applications. Moreover, PBEVs exhibit natural resistance to digestive enzymes, which enhances their potential for drug delivery via the gastrointestinal tract, unlike mammalian EVs that degrade quickly in biological fluids [103].

#### 4.1.2. Storage-Related Stability Challenges

Freeze-thaw cycles can lead to the rupture of vesicular membranes, the release of cargo, and vesicle aggregation [104]. This phenomenon is due to the formation of ice crystals, which puncture lipid membranes. Guarro et al., demonstrated that ginger-derived vesicles underwent significant structural damage after five freeze-thaw cycles, with a reduction in drug encapsulation efficiency. This study also highlighted that using cryoprotectants like sucrose and trehalose during freeze minimized vesicle damage by preventing ice crystal formation [105].

Lyophilization is an effective strategy to extend the shelf-life of PBEVs. However, the process can induce changes in vesicle morphology, which may affect their drug-carrying ability. Cryoprotectants, such as mannitol, sucrose, or trehalose, are typically added during lyophilization to stabilize the vesicle structure. In an interesting study, a group of researchers showed that turmeric-derived vesicles lyophilized with cryoprotectants exhibited minimal size changes upon rehydration and maintained their drug encapsulation efficiency for over 6 months, compared to vesicles lyophilized without cryoprotectants, which showed significant aggregation and cargo loss [104]. While lyophilization is commonly used for stabilizing PBEVs, animal-derived EVs require more stringent conditions, often needing additional lipid-stabilizing agents to maintain their bioactivity post-lyophilization. Similarly, bacterial EVs exhibit better resilience against freeze-thaw cycles and drying due to their robust membrane composition, making them more naturally suited for long-term storage compared to both plant and mammalian EVs.

#### 4.1.3. Effect of Formulation Components

The buffer where PBEVs are suspended can play a major role in maintaining their stability. PBS is frequently used, but its high ionic strength can promote vesicle aggregation by reducing electrostatic repulsion. In contrast, Walker et al., investigated EVs and found that those stored in isotonic sucrose buffer exhibited better stability (in terms of size and zeta potential) over three months, compared to those stored in PBS, which showed a reduction in vesicle stability due to aggregation [106]. In contrast, animal-derived EVs often require complex storage buffers containing proteins and stabilizers such as albumin to prevent aggregation and degradation [107], while bacterial EVs, due to their robust outer membrane composition, show better stability even in minimal buffer conditions. These differences in formulation requirements highlight the need for tailored storage strategies depending on the EV source and intended application.

### 4.2. Techniques for Evaluating the Stability of PBEVs

**Figure 3:**
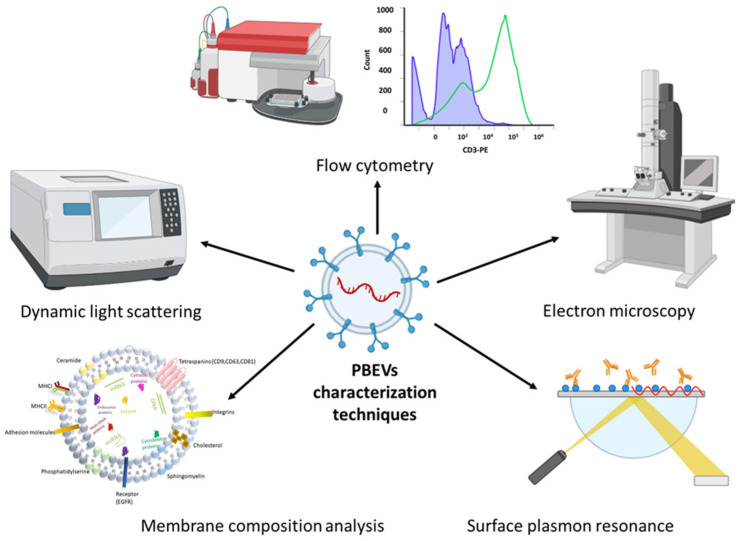
Most commonly used characterization techniques for PBEVs.

Understanding the specific traits of individual EV samples is crucial for fully harnessing their potential as therapeutic carriers. Characterization poses a challenge due to the considerable variation in EV samples, particularly in their intravesicular content and membrane composition. While literature over the past decade has made significant strides in characterizing EVs, future research is needed to thoroughly define their intrinsic contents, surface markers, and biological functions. Such comprehensive characterization is essential before EVs can be approved as therapeutic carriers in clinical settings. For PBEVs, complete characterization will be pivotal in determining their applicability in biomedical and pharmaceutical research. This section discusses commonly used methods for characterizing EVs, as illustrated in Figure 3. To address the challenges of characterizing nano-sized EVs, especially without relying on specific markers, researchers often employ combination methods that integrate both optical and non-optical techniques. These combined approaches enhance the accuracy and depth of EV characterization, helping to elucidate their structure, composition, and functional properties.

#### 4.2.1. Morphological Characterization Methods

The morphological characterization of EVs is crucial for understanding the applications and functions of EVs derived from different plant sources or parts. The differential composition of EVs from various parts of the same plant can lead to significant morphological differences, which can impact their functionality. Therefore, it is essential to visualize the morphology of EVs using sophisticated techniques such as electron microscopy (EM) and atomic force microscopy (AFM). These advanced imaging techniques allow for a detailed assessment of EXO shape, size, and structural integrity, which are key factors in their potential therapeutic use. Moreover, morphological characterization can aid in selecting the most appropriate separation technique for isolating EVs, ensuring that the integrity of these vesicles is preserved. Since EVs often undergo aggregation during the separation process, morphological evaluation is also valuable in detecting and addressing such issues, thereby optimizing the quality and consistency of the isolated EVs [108,109,110].

##### EM for Structural Analysis

EM is a widely used technique for analyzing the size and structure of EVs. In this method, concentrated EVs are typically fixed onto grids or stages, stained with dyes, and then visualized using various forms of EM, such as TEM and scanning electron microscopy (SEM) [111]. The images obtained from EM provide detailed information about the size, morphology, and, when appropriately labeled, the presence of specific biomarkers on the EVs.

However, EM has some limitations. One key drawback is that it does not allow for the measurement of EV concentration. Additionally, the process of preparing EVs for EM is complex and time-consuming, requiring steps like dehydration, fixation, and metallization. These procedures can alter the native state of the EVs. To address this, cryo-electron microscopy (cryo-EM) offers a significant advantage, as it allows for the direct imaging of single vesicles in a state that is much closer to their natural, hydrated form [108], thereby preserving their native structure and minimizing artifacts introduced by processing.

##### AFM for Surface Characterization

AFM offers a powerful tool for obtaining detailed 3D micrographs of individual EVs without the need for staining or fixation. In AFM, a probe scans the surface topography of EVs, allowing for the visualization of the intricate nanostructure of the EXO surface with high resolution. Unlike SEM and TEM, which require dried and often deformed samples, AFM can observe EVs in their native state using in-liquid imaging techniques [109,112]. This capability is particularly valuable for gaining a full understanding of the EXO structure and how it correlates to their properties and stability in bulk solutions. AFM’s ability to maintain the native structure of EVs is crucial for accurately assessing their behavior in biological environments, which is essential for the formulation development process. The primary limitation of AFM is the high cost of the equipment, which can be a barrier for some research facilities. However, its advantages in providing detailed and native-state imaging make it a highly useful technique for EXO characterization.

#### 4.2.2. Size Distribution and Surface Charge Analysis

##### Dynamic Light Scattering (DLS) and Nanoparticle Tracking Analysis (NTA)

The size of EVs is a critical factor in their effectiveness for drug delivery applications. Consistency in the size of EVs extracted from various plant sources is important to ensure uniformity in their therapeutic potential. Additionally, the zeta potential, which reflects the surface charge of EVs, plays a vital role in their stability. Variations in the composition of EVs can alter their surface charges, potentially leading to aggregation in solution. Therefore, analyzing both the size and zeta potential of EVs is essential not only for selecting the appropriate isolation method but also for ensuring maximum stability [113]. To accurately assess the size and concentration of single EVs, new light scattering-based techniques have been developed, including dynamic light scattering (DLS) and nanoparticle tracking analysis (NTA) [114,115]. DLS measures the size distribution of EVs by analyzing the dynamic fluctuations in light scattering caused by the Brownian motion of particles. Since the diffusion rate of EVs is temperature-dependent, careful sample preparation is crucial for obtaining accurate DLS measurements. However, DLS is less suitable for heterogeneous mixtures of EVs, as the presence of larger particles can significantly reduce the accuracy of the measurements.

NTA, on the other hand, combines dark-field microscopy with motion-tracking software to quantify the concentration and size distribution of EVs. The technique calculates the diameter of EVs using the Stokes-Einstein formula. NTA can also be used to profile fluorescently tagged EVs, providing additional insights into their properties. Despite its advantages, NTA has limitations; it cannot differentiate vesicles with identical sizes and Brownian motions, which necessitates thorough isolation and purification procedures before analysis. Currently, NTA is the most frequently employed method for measuring the concentration and size distribution of EVs, making it a valuable tool in the characterization of EVs for drug delivery and other applications [112,114].

##### Flow Cytometry for EV Profiling

EVs in fluids can be effectively characterized using flow cytometry, a laser-based technique that provides detailed information about their chemical and physical properties. Typically, flow cytometry can identify vesicles larger than 300 nm in diameter. However, due to the weak signals emitted by smaller EVs, analyzing these particles can be challenging. To address this limitation, EVs are often coupled with larger beads and labeled with a fluorophore, which enhances the detection capabilities of flow cytometry. This technique is highly effective for measuring and analyzing individual EVs, including multiple surface markers on each EXO [116]. POL et al. developed a method for determining vesicles using flow cytometry through two detection techniques: single and swarm detection [117]. The single detection method allows for the detection and estimation of vesicles with diameters between 300 and 700 nm. In contrast, the swarm detection mode enables the detection of vesicles smaller than 300 nm. This mode works by counting the scattered laser beam from multiple small vesicles as a single event signal. Combining single and swarm detection techniques is particularly useful for analyzing polydisperse samples, where vesicles of varying sizes are present. The research also emphasized the importance of proper calibration and the selection of appropriate detector types in the flow cytometer to accurately determine the size of EVs. These advancements in flow cytometry have improved the accuracy and reliability of EXO characterization, making it a valuable tool for studying EVs in various biological and clinical applications.

#### 4.2.3. Emerging Technologies for PBEV Characterization

##### Single-Particle Analysis Techniques

One such advanced technique is single-particle analysis, which allows for high-resolution imaging and quantitative assessment of individual EVs [118]. Unlike bulk analysis methods, single-particle techniques, such as nanoflow cytometry and high-resolution microscopy, enable researchers to distinguish subpopulations within EV samples, offering valuable insights into their heterogeneity [119].

##### Super-Resolution Microscopy for Nanoscale Imaging

Super-resolution microscopy, such as stimulated emission depletion (STED) microscopy and structured illumination microscopy (SIM), has also gained traction in EV research. These techniques surpass the resolution limits of conventional fluorescence microscopy, enabling detailed visualization of EV morphology and molecular composition at the nanoscale [22,120].

##### Surface-Enhanced Raman Spectroscopy (SERS) for Molecular Profiling

Another emerging approach is Raman spectroscopy and surface-enhanced Raman spectroscopy (SERS), which provide label-free molecular fingerprinting of EVs, offering detailed insights into their biochemical composition [119,121]. It is a non-image-based technique that can be used to elucidate the peptide and nucleic acid composition to understand the structure of the EVs.

##### Microfluidic-Based EV Characterization

Microfluidic-based characterization is rapidly evolving as a promising alternative to traditional bulk analysis. Microfluidic platforms, often integrated with dielectrophoresis or nanopore sensing, enable real-time, high-throughput analysis of individual EVs, facilitating their size, charge, and molecular profiling in a single step [122]. Such systems enhance the efficiency and accuracy of EV characterization while minimizing sample requirements.

##### Tunable Resistive Pulse Sensing (TRPS) for Size and Concentration Analysis

TRPS is an emerging technique that offers precise size distribution and concentration measurements of EVs by detecting changes in electrical resistance as vesicles pass through a nanopore [22]. This method provides a highly sensitive approach to characterizing PBEVs [123], especially when used alongside other analytical techniques.

#### 4.2.4. Study of Molecular Interactions by Surface Plasmon Resonance

Nanosensors based on surface plasmon resonance (SPR) are rapidly advancing as tools to explore interactions between ligands and biomolecules, making them particularly suitable for EV detection. SPR-based Nanosensors are advantageous because they are label-free and allow for the real-time, quantitative measurement of binding affinities. When EVs bind to a surface, the adsorbed layer’s thickness increases, leading to changes in the refractive index, which can be detected by SPR [124]. Im et al. developed a nano-plasmonic sensor called nano-plasmonic EXO (nPLEX) specifically for identifying and profiling EXOs [125]. In this system, target-specific ligands are attached to periodic nanohole arrays within the nPLEX sensor, designed to trap EXOs. The nanoholes’ diameter and periodicity are finely tuned to match the average diameter of EXOs. When an EXO binds to the nanohole array, it causes a spectrum shift proportional to the mass of the EXO bound to the array. This system is further enhanced with miniaturized optics, including a laser diode and an image sensor, to monitor changes in transmitted light intensities, allowing for parallel measurements. Similarly, Rupert et al. employed SPR by immobilizing probes, such as antibodies, on a surface designed to capture EXOs for concentration determination [126]. The extent of EXO coverage on the surface correlates with the concentration of specific EXOs in the solution. However, this method faces some challenges, such as the variable and complex surface composition of EXOs, which can affect detection accuracy. The effectiveness of detection depends significantly on the specific surface modification techniques employed, making it essential to carefully design and optimize these systems for reliable EXO analysis.

According to the above-cited literature, a combination of techniques provides the most comprehensive characterization of PBEVs. TEM and AFM remain the gold standards for morphological analysis due to their ability to capture high-resolution images. For size distribution and surface charge analysis, NTA and DLS are widely used, with NTA offering more precise single-vesicle measurements. Flow cytometry, when optimized with fluorescence labeling, allows for high-throughput EV quantification and marker profiling. Emerging methods such as microfluidic-based analysis and SERS provide real-time, high-throughput analysis, making them valuable additions to conventional techniques. Therefore, a multi-model approach, integrating TEM or AFM for morphology, NTA for size and concentration, and flow cytometry for biomarker profiling, is considered the most robust characterization strategy for PBEVs.

#### 4.2.5. Membrane Composition Analysis

Characterizing membrane composition is essential for quality control and maximizing the therapeutic potential of EVs. The composition of PBEVs can vary significantly depending on their plant source. These EVs contain a diverse array of biomolecular components, including proteins, nucleic acids, lipids, and metabolites such as alkaloids, glycosides, and flavonoids. These components play crucial roles in immune recognition, EXO mobility, and cell signaling. To separate these components, techniques such as gel electrophoresis, gas chromatography, and high-performance liquid chromatography (HPLC) can be employed. For identification purposes, methods like polymerase chain reaction (PCR), mass spectrometry (MS), nuclear magnetic resonance (NMR), and fluorometric assays are utilized. For example, Hegmans et al. established a protocol combining 1-D gel electrophoresis (SDS-PAGE) with MALDI-TOF mass spectrometry to identify EXO proteins [127]. Mears et al. used 2-D gel electrophoresis (SDS-PAGE) in conjunction with mass spectrometry for similar identification tasks [128].

**Figure 4:**
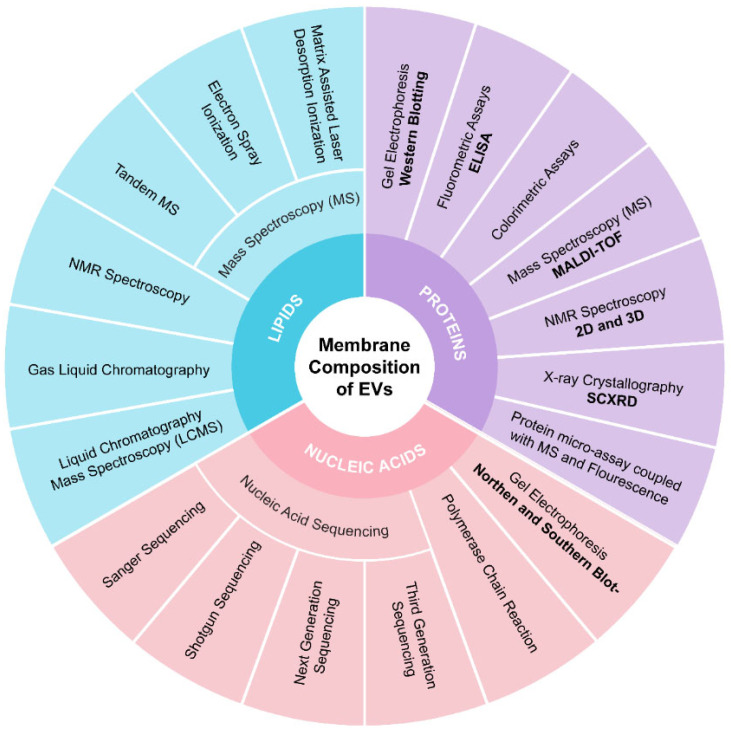
Analytical methods for identifying and quantifying the EVs membrane composition.

Western blotting is a widely used method for detecting specific proteins in EXO membranes [129]. For a comprehensive analysis, proteomic techniques such as SDS-PAGE or HPLC followed by mass spectrometry can be used. Conde-Vancells et al. provided a detailed description of the protein composition of hepatocyte-derived EXOs using these methods [130]. Lipidomics is also critical for studying EXO lipid profiles. Llorente et al. demonstrated variations in the lipid composition of cells and their EXOs through extensive lipid analysis [131]. The most recent data on protein and lipid compositions related to EXOs are compiled in various databases, including Vesiclepedia. For nucleic acid characterization, techniques such as northern and southern blotting, PCR, and sequencing methods (e.g., Sanger sequencing, next-generation sequencing) are employed. Databases like miRbase, Kyoto Encyclopaedia of Genes and Genomes (KEGG), and Gene Ontology (GO) are useful for identifying nucleic acids obtained from PBEVs. Figure 4 outlines the analytical methods useful for analyzing EVs membrane composition.

#### 4.2.6. Advanced Computational Approaches in EV Stability Analysis

The application of artificial intelligence (AI) and machine learning in EV research has revolutionized the characterization and predictive analysis of EVs [132]. AI-driven image processing algorithms can efficiently analyze EV morphology, size distribution, and aggregation patterns, providing higher accuracy than traditional methods. Machine learning models are also being developed to predict EV stability under different storage conditions by analyzing physicochemical parameters and degradation kinetics. Recent studies have utilized deep learning approaches to automate EV classification, distinguish between different vesicle subtypes, and identify biomarkers within their cargo [133]. The integration of AI and ML in EV characterization will significantly improve the reproducibility and scalability of PBEV-based therapeutic applications, ensuring better stability profiling and enhanced quality control.

## 5. Strategies to Enhance the Stability of PBEVs

The natural stability of PBEVs can be insufficient for clinical applications, especially when they are required to deliver therapeutic agents in harsh physiological environments. Therefore, several strategies have been developed to enhance the stability of PBEVs. These strategies include chemical and physical modifications, encapsulation techniques, and the use of stabilizing agents. This section discusses the most relevant methods to improve PBEV stability and ensure functional integrity during drug delivery.

### 5.1. Chemical and Physical Modifications for Stability Improvement

Polyethylene glycol (PEGylation) involves the attachment of PEG chains to the surface of vesicles. This modification prevents opsonization, the process by which immune cells identify and clear foreign particles, and reduces vesicle aggregation by increasing surface hydration [104]. Zhang et al. [134] reported the PEGylation of lemon-derived EVs increased their circulation time in vivo in mice models, which led to a higher accumulation in tumor tissues when used for anticancer drug delivery. PEGylation also improved the vesicle stability in varying pH conditions. For example, PEGylated ginger EVs remained stable in pH 5 conditions for 48 h, compared to non-PEGylated vesicles that began to aggregate within 24 h.

Incorporating additional lipids, such as cholesterol or sphingomyelin, into PBEV membranes enhances membrane rigidity and reduces permeability. Li et al. [85] demonstrated that ginger-derived EVs, when loaded with folic acid-modified and cholesterol-coupled siRNA, exhibited improved drug loading (up to 80%). Cholesterol conjugation improved the stability of siRNA along with enhancing its membrane interaction. Cholesterol conjugation also improved the cellular uptake of vesicles. Folic acid ligand promoted the uptake of vesicles by epidermal cells. The conjugation significantly improved drug delivery outcomes against cancer growth.

### 5.2. Encapsulation Techniques for Preservation

Encapsulating PBEVs in biocompatible polymers, such as poly(lactic-co-glycolic acid) PLGA or chitosan, provides an additional layer of protection, preventing degradation and enhancing stability [135]. Zhang et al. [136] encapsulated ginger-derived 6-shagaol in PLGA nanoparticles and found that the encapsulated vesicles had much better biocompatibility compared to non-modified vesicles. The encapsulated EVs delivered 6-shogaol with an enhanced efficiency compared to non-encapsulated controls, highlighting the potential of polymer-based encapsulation for oral drug delivery systems. Layer-by-layer assembly is another technique used to enhance vesicle stability. This method involves sequential deposition of oppositely charged polymers or molecules onto the vesicle surface, creating a multi-layered structure that enhances mechanical stability, creating a multi-layered structure that enhances mechanical stability and prolongs circulation time. Deng et al. [137] applied LbL assembly to mesenchymal stem cell-derived EVs (MSC-EXOs) using alternating layers of N-(2-hydroxyl) propyl-3-trimethyl ammonium chitosan chloride (HTCC) and oxidized konjac glucomannan (OKGM) polysaccharides. The modified vesicles showed controlled release in the inflammatory colon to exert anti-inflammatory and tissue repair effects.

### 5.3. Lyophilization and Freeze-Drying Approaches

EVs, including EXOs and microvesicles, have gained significant attention as potential therapeutic and diagnostic agents due to their role in intercellular communication. However, their clinical translation is hindered by stability concerns, as EVs are highly susceptible to degradation, aggregation, and loss of bioactivity during storage. Lyophilization has emerged as a promising technique to enhance the stability of EVs by removing water content while preserving their structural integrity and functionality [138].

Lyophilization involves three key stages: freezing, primary drying (sublimation), and secondary drying (desorption). In the freezing stage, EV samples are rapidly cooled to form ice crystals, which are then removed via sublimation under vacuum conditions in the primary drying phase. The final secondary drying step eliminates any residual moisture, ensuring long-term stability [139]. This process prevents hydrolytic degradation and limits oxidative damage, both of which can compromise EV integrity in liquid storage. One of the major advantages of lyophilization is the ability to store EVs in a dry powder form, significantly extending their shelf life at room temperature or under refrigerated conditions. Unlike fresh EV preparations, which often require ultralow-temperature storage (−80 °C or liquid nitrogen) to maintain their bioactivity, lyophilized EVs remain stable for extended periods without substantial loss of functional components [138]. Additionally, the reduced reliance on deep freezing minimizes logistical challenges and costs associated with EV transport and storage, making lyophilization an attractive option for large-scale production and clinical applications.

Despite its benefits, the lyophilization process can impose stress on EV membranes, potentially affecting their morphology and cargo retention. To mitigate these risks, stabilizing agents such as cryoprotectants and antioxidants are commonly incorporated into the formulation. Cryoprotectants like trehalose and mannitol have been widely used to stabilize vesicles during freezing and lyophilization. These sugars prevent the formation of ice crystals that can damage vesicle membranes during the freezing process. Kim et al., [98] showed that EVs derived from leaves of *Dendropanax morbifera* and TMO maintained their size and cellular uptake with changes in time and temperature after a number of freeze-thaw cycles, compared to a significant reduction in stability for vesicles freeze-thawed without cryoprotectants. In addition to cryoprotectants, oxidative damage to vesicles can be mitigated using antioxidants such as glutathione or vitamin E [140]. Antioxidants help prevent lipid peroxidation and maintain vesicle membrane integrity. Biscans et al., [141] found that the vitamin E and siRNA conjugation yielded better loading efficiency of siRNA into EVs with increased RNA delivery to neurons.

Several comparative studies have highlighted the superiority of lyophilization over conventional storage methods. For instance, EVs stored at −80 °C for extended periods often experience aggregation and loss of cargo stability, whereas lyophilized EVs maintain their structural integrity and functional properties. Additionally, lyophilization enables the reconstitution of EVs in various buffers or media without significant alterations in particle size or zeta potential. Furthermore, lyophilization provides an advantage in the formulation of EV-based pharmaceuticals, where controlled release delivery systems can be developed using lyophilized EVs incorporated into hydrogels, nanoparticles, or lyophilized powders for reconstitution. This approach facilitates precise dosing and enhances the practicality of EV-based therapeutics in clinical settings.

While lyophilization presents a promising strategy for EV preservation, further optimization is needed to refine the process parameters for different EV subtypes. Factors such as freezing rate, drying time, and stabilizing agent selection must be tailored to preserve the unique biochemical composition of EVs derived from diverse sources, including plant, bacterial, and mammalian cells. Additionally, regulatory considerations must be addressed to ensure the quality, potency, and reproducibility of lyophilized EV products for therapeutic applications. Overall, lyophilization represents a transformative approach to improving the stability and scalability of EV-based therapeutics. Continued advancements in this field will pave the way for the widespread adoption of EVs in biomedical research and clinical medicine.

## 6. Quality Control Parameters for PBEVs

Before advancing to clinical trials, it is essential to meet specific quality control parameters for EVs. As the field is still evolving, ongoing research and comprehensive studies are needed to refine these parameters. However, it is crucial to establish standards for the content and composition of therapeutic vesicles. A major challenge in this domain is the lack of universally accepted guidelines, necessitating the development of standard operating procedures for isolation, purification, and quality assessment of PBEVs. This section provides an overview of the current findings related to the intravesicular contents and membrane composition of EVs.

### 6.1. Characterization of Intravesicular Contents

Previous studies have demonstrated that both EXOs and microvesicles carry transcription factors and RNAs originating from their parent cells [142,143]. However, their molecular content does not always accurately reflect the profile of the source cells. EVs function as carriers of intracellular components, facilitating communication between cells. Interestingly, they are often enriched with specific proteins and RNAs, although the mechanisms governing this selective packaging and enrichment remain largely unclear [144]. Moreover, several reports highlight that PBEVs exhibit unique bioactive molecules compared to mammalian EVs, such as plant-specific miRNAs, flavonoids, and polysaccharides, which could provide additional therapeutic advantages [103]. Research on cancer-associated EVs has grown substantially, with increasing evidence suggesting that EVs derived from malignant cells contribute to metastasis. For example, a study by Skog et al. demonstrated that glioblastoma-derived microvesicles, which carry RNA and pro-angiogenic proteins such as angiogenin, VEGF, and TIMP-2, can promote both metastasis and angiogenesis [145]. Conversely, Baj-Kryzworzeka et al. found that microvesicles from tumor cells carrying vascular endothelial growth factor (VEGF), hepatocyte growth factor (HGF), and interleukin-8 (IL-8) exert anti-apoptotic effects on monocytes, suggesting that these cancer-derived vesicles support macrophage survival [146]. While these findings underscore the importance of considering the intrinsic biological functions of EVs when designing therapeutic carriers, comparative evaluations between PBEVs and mammalian EVs in disease models remain limited. Future research should focus on systematically characterizing the bioactive cargo of PBEVs to determine their potential therapeutic implications.

### 6.2. Membrane Composition and Structural Integrity

EVs express certain surface markers and play an essential role in their membrane composition as they possess biological functions. EVs are generally characterized by the presence of enriched transmembrane proteins such as CD9 and CD63, along with integral membrane proteins like Flotillin-1 [147]. Additionally, EVs display surface molecules that reflect the specific cell type and activation state of their origin. For instance, cancer-derived exosomes (EXOs) often express the NKG2D ligand, which can interact with T and B cells as a mechanism of immune evasion. While the surface marker composition of EVs is influenced by their parent cell, it frequently differs significantly from that of the original plasma membrane. In contrast, MVs retain the surface composition of the plasma membrane, as they are formed by direct outward budding from the plasma membrane [143].

Unlike mammalian EVs, PBEVs contain plant-specific lipid profiles, including phospholipids and sterols, which influence their stability and bioavailability. Some studies suggest that the lipid composition of PBEVs could improve their resistance to enzymatic degradation, potentially extending their circulation time in biological systems [103]. However, additional comparative studies are needed to establish their stability profiles under physiological conditions.

One of the most compelling aspects of employing extracellular vesicles (EVs) for therapeutic delivery lies in their potential for innate targeting, immune activation, and immune evasion. Studies have reported that EVs can selectively target specific cell types based on their cellular origin; however, the exact mechanisms governing these interactions remain to be fully elucidated. In 2006, Obregon et al. demonstrated that EVs possess immune-activating capabilities [148]. Their study showed that microvesicles derived from lipopolysaccharide-stimulated dendritic cells could prime T-cells by transferring antigenic material to resting dendritic cells. Furthermore, EVs originating from cells expressing major histocompatibility complex (MHC) markers were found to carry both MHC class I and II proteins. These surface molecules allow EVs to transfer cargo within the extracellular space while avoiding immune detection and clearance.

While mammalian EVs have been extensively studied for their immunomodulatory functions, the immunogenicity of plant-based extracellular vesicles (PBEVs) remains less understood. Preliminary findings suggest that PBEVs may have lower immunogenic potential compared to their mammalian counterparts, positioning them as promising candidates for drug delivery without eliciting strong immune responses [97]. Nevertheless, comprehensive in vivo investigations are necessary to confirm these findings and ensure the long-term safety of PBEV-based therapeutics.

Future research should aim to unravel the intrinsic functions of EVs and how these are influenced by their molecular composition. A deeper understanding of these relationships will be critical to advancing EVs as precision tools for targeted drug delivery, immunotherapy, and immune evasion strategies in clinical applications.

## 7. Potential of PBEVs in Nucleic Acid Delivery and Associated Stability Challenges

EVs hold significant promise as drug delivery systems due to their natural ability to transport materials between cells. They can be engineered to enhance uptake or loaded with specific cargo for targeted distribution. While previous research has demonstrated the potential of EVs for drug delivery, several challenges must be addressed before EV technology can be used clinically. Since the properties of EVs are influenced by their production conditions and the cell of origin, establishing precise characterization procedures is crucial to ensure safety and reproducibility. Additionally, developing methods to produce EVs in large quantities is essential to meet the high-yield requirements for clinical trials. The protective bilayer membrane of EVs offers stability and shields nucleic acids from enzymatic degradation, making them ideal candidates for nucleic acid delivery. Their low immunogenicity also allows for the possibility of multiple injections [34]. Edible plants present a promising and cost-effective source for large-scale EV production. EVs can be efficiently recovered through scalable methods and are particularly abundant in the juice of various edible plants. Given that these PBEVs are non-immunogenic and harmless, as they are naturally consumed in fruits and vegetables, they are strong candidates for oral administration. Several studies have explored techniques for loading nucleic acids into PBEVs, demonstrating that these EVs can effectively carry nucleic acids in an intact and functional form, while protecting from enzymatic degradation [113,114].

**Figure 5:**
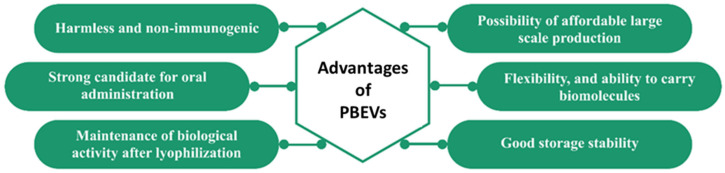
Numerous advantages offered by PBEVs.

PBEVs offer numerous advantages, as highlighted in Figure 5. The methods for loading nucleic acids into PBEVs involve several steps, including electroporation, sonication, passive internalization, and chemical or mechanical permeabilization. Electroporation creates transient pores that allow nucleic acids to enter EVs, while sonication modifies membrane structures in the presence of nucleic acids. Passive internalization of nucleic acids occurs under specific pH, temperature, and salinity conditions. Chemical or mechanical permeabilization methods also increase membrane permeability, facilitating nucleic acid entry. The effectiveness of these methods can vary depending on the type of nucleic acid and the source of the EVs. In drug delivery systems, the goal is to deliver a pharmaceutical molecule to achieve its desired therapeutic effect with minimal side effects. However, issues such as poor pharmacokinetics, loss of tissue selectivity, immunogenicity, increased systemic clearance, and toxicity are often observed in current dosage forms available on the market. EVs have the potential to address these challenges due to their natural origin, small size, flexibility, surface molecules, and ability to carry biomolecules. Several EV-based medications are currently under investigation. Despite the recognition of EVs’ significant potential for targeted drug delivery in cancer [75], hepatitis C, neurological disorders [149], inflammatory conditions, and more, this research is still in its early stages. EVs from natural sources, including cell cultures and bodily fluids, present challenges related to the heterogeneity of the vesicle population and isolation techniques, which can affect the composition and characteristics of the vesicles.

Pomatto et al. observed that edible PBEVs could be suitable carriers for delivering various therapeutic molecules, particularly for mucosal absorption [150]. The impact of storage conditions on EVs has been extensively studied, revealing that storage-related factors can affect EV characteristics such as biophysical stability, particle recovery and size, and overall function [107]. Phosphate-buffered saline (PBS) has been the preferred storage buffer in many studies, with recommendations to store EV aliquots at −80 °C. However, recent research has also explored the effects of storage temperature and time, the role of plastic tubes in isolation or storage, and the use of cryoprotective additives like DMSO or trehalose.

More recent studies have investigated the stability of EVs in the context of lyophilization, particle loss through vesicle fusion during storage, and the short-term effects of storage conditions and concentration methods on EV recovery and function. However, findings have sometimes been conflicting, often due to the focus on specific biofluid samples or EV isolation methods, which can influence EV composition, recovery, and purity. The lack of consensus on the impact of storage conditions on EVs highlights the need for comprehensive studies that systematically compare different storage strategies over extended periods. This is especially important for stabilizing purified EV preparations, particularly those derived from cell culture supernatants [107]. In their study, Pomatto et al. investigated the potential of EVs derived from the juice of *Citrus sinensis* (orange) as carriers for an orally administered mRNA vaccine coding for the S1 protein subunit of SARS-CoV-2 [150]. Remarkably, the mRNA loaded into these PBEVs remained protected and stable at room temperature for a year after lyophilization and encapsulation [151]. To preserve the biological activities of PBEVs and facilitate their clinical use, it is crucial to maintain proper storage conditions. Plant extracts, if not properly stored before isolating PBEVs, could negatively impact the function, content, and separation of these vesicles. Although freezing temperatures, typically at −80 °C or 4 °C, are commonly used in laboratories today, these conditions may not be ideal for maintaining the integrity of isolated EVs, as storage can lead to changes in EV content, function, size, and shape [152].

Cryopreservation is a method that applies low temperatures (−196 °C, −80 °C, or −4 °C) to preserve the functional stability of biological particles, including PBEVs [153].

Freeze-drying is another technique for storing EVs, including PDENs. This method involves freezing moisture-containing materials below their freezing point and then combining sublimation and desorption to preserve them. However, freeze-drying can damage the molecular structure of biomolecules due to the stresses of freezing and dehydration [154]. Dry particles are produced by rapidly vaporizing PDEN solution after it has been atomized in a drying room and interacted with hot air [39]. Several variables, including the EV solution supply, the atomization pressure, and the output temperature, might impact the stability [155]. Andaloussi et al. examined the effects of various storage buffer formulations at different temperatures on EVs from diverse cellular sources over two years [107]. Their study evaluated EV parameters such as concentration, diameter, surface protein profile, and nucleic acid content. They found that EVs stored in PBS, particularly pure samples at all temperatures studied, showed significantly reduced recovery within days. The use of PBS as a diluent also rapidly decreased EV recovery rates. However, supplementing PBS with trehalose and human albumin (PBS-HAT) significantly mitigated these effects, resulting in improved EV recovery, stability over multiple freeze-thaw cycles, and enhanced short- and long-term preservation of EV samples stored at −80 °C [107]. Table 3 summarizes the stability studies conducted on EVs derived from both plant and animal sources, highlighting the importance of optimized storage conditions for maintaining EV integrity and function.

## 8. Conclusions and Future Perspective

Despite significant advancements in EV-based research, no EV-based product has yet been approved by the US FDA. This delay can be attributed to the complexity of the manufacturing processes and the stringent requirements for process monitoring. EVs, particularly those modified to contain specific bioactive molecules such as mRNA or miRNA, hold promise for inducing targeted responses in humans to improve disease conditions. However, the preclinical investigations that have predominantly utilized human-derived EVs isolated from cell culture-conditioned media face substantial challenges for clinical applications. These challenges include high costs and low productivity, largely due to the necessity for strict adherence to good manufacturing practice (GMP) conditions throughout the entire cell culture process and the requirement for rigorous sterile purification standards. In response to these challenges, the focus has shifted towards PBEVs as a potential alternative for industrial use. Unlike human-derived EVs, PBEVs do not require cell cultivation since they are already present in nature [9,151], particularly in extractive products such as plant juices. For example, EVs extracted from the juice of edible plants like oranges offer a cost-effective solution due to their natural abundance. Recent studies have demonstrated that PBEVs are enriched with plant-specific bioactive molecules such as flavonoids, polyphenols, and plant miRNAs, which have shown promising immunomodulatory and therapeutic effects. One of the significant advantages of edible EVs is their potential for oral administration, which can promote mucosal immunity—a crucial first line of defense at the point of virus entry. These EVs are particularly well-suited for drug delivery because they protect nucleic acids from environmental stressors and enzymatic degradation. Their native membrane envelope facilitates the delivery of therapeutic payloads and enhances their entry into target cells. Compared to synthetic delivery systems such as lipid nanoparticles (LNPs), synthetic lipoparticles, and adenoviruses, PBEVs offer several benefits. They are non-cytotoxic, biocompatible, and possess an ideal safety profile since they are natural substances and dietary components. Moreover, their resilience to stress enables lyophilization and storage at room temperature, which further adds to their practicality for large-scale applications. This characteristic makes them particularly attractive for global distribution, especially in low-resource settings where cold chain storage may not be feasible.

However, preserving the biological activities of PBEVs during storage remains a critical challenge. The storage conditions of plant extracts before isolating PBEVs can significantly impact their function, content, and separation, similar to challenges faced with biofluid samples. While freezing temperatures, typically at −80 °C or lower, are commonly used in laboratory settings, these conditions may not be optimal for maintaining the integrity of isolated EVs. Storage at these temperatures can lead to changes in EV content, function, size, and shape, highlighting the importance of finding storage solutions that preserve their biological activity. Emerging stabilization techniques, including cryoprotectants, lyophilization with protective excipients, and encapsulation within hydrogel matrices, have shown potential to maintain the functional stability of EVs during storage and transportation.

Furthermore, the future industrial scalability of EV-based products will require overcoming key challenges in large-scale production, standardization, and regulatory approval. One major bottleneck is the lack of well-defined, cost-effective isolation and purification techniques that can be efficiently applied in industrial settings. Advances in bioprocess engineering, such as high-throughput EV isolation methods and scalable bioreactor-based production, will be crucial for making EV-based therapeutics viable for commercial use. For instance, recent advancements in tangential flow filtration (TFF) and size-exclusion chromatography (SEC) have demonstrated improved efficiency in EV purification while maintaining high yield and bioactivity. Additionally, regulatory bodies such as the US FDA and EMA need to establish standardized guidelines for EV characterization, safety, and efficacy to streamline their clinical translation. Addressing these challenges will be essential in unlocking the full potential of EVs in diagnostics, therapeutics, and drug delivery applications.

Despite these challenges, it is anticipated that the production and storage of drug-loaded PBEVs will pose fewer difficulties compared to the production and storage of cell culture-based EVs. The natural resilience of PBEVs, combined with their ability to be stored at room temperature, offers a promising avenue for overcoming the obstacles that have hindered the clinical translation of EV-based therapies. Future research should focus on optimizing large-scale isolation methods, investigating the stability of PBEVs under various storage conditions, and conducting rigorous in vivo studies to validate their therapeutic efficacy in different disease models.

## Figures and Tables

**Table 2:** PBEVs like nanoparticles application in treating diseases and challenges.

Tissue/Disease for Targeting	Prospects	Challenges and Issues	PDENs Source	Findings	Ref.
Periodontitis	PBEVs have the potential to transport drugs for oral mucosal delivery to regulate oral immunity to periodontopathogen	PBEVs are limited to carry minimal amounts of drugs, with an unclear mechanism of cellular uptake as it may differ with each extraction batch	Citrus sinensis Ginger	PBEVs protect their miRNA contents from degradation by human salivary enzymes to act as a delivery agent PBEVs are absorbed by *P. gingivalis* and contribute to the inhibition of bacterial growth through their interaction with HBP35 and membrane depolarization, preventing bacterial attachment to oral epithelial cells and periodontal bone loss	[80,81,82]
Colitis, tumors, liver diseases, skin diseases.	PBEVs have the potential to act as a drug-delivery system for the delivery of RNAs and lipids to inhibit the inflammation genes, as well as bacterial and tumor growth	There is a lack of transparency on quality control and evaluation systems, stability, biomarker confirmation, and biochemical characterization	Shiitake mushroom, Lemon, Broccoli Ginger roots Blueberry, coconut, ginger, grape-fruit, Hami melon, kiwifruit, orange, pea, pear, soybean, and tomato	PBEVs mitigate the NLRP3 activation by inhibiting Casp1 p10 level, and IL-1β protein assembly by significantly decreasing the pro-IL-1β, leading to liver protection The modification of PBEVs with arrow tail RNA enabled the delivery to and targeting of specific cells with a significant reduction in surviving expression and tumor growth PBEVs, which consist of miRNAs, can interact with mammalian mRNAs of inflammatory mediators directly	[83,84,85]
Unspecified	Simplified large-scale mass production for their large biodiversity with minimal cytotoxicity for drug-delivery system	The internalization mechanisms remain elusive, and there is a lack of clarity regarding their specific receptors and ligands for PBEVs. Biosafety and toxicity of genetic transfer are unclear	*Citrus sinensis* In vitro Ginger roots	PBEVs protect their miRNA contents from degradation by human salivary enzymes acting as an effective delivery agent. The modification of PBEVs with arrow tail RNA enabled the delivery to and targeting of specific cells with a significant reduction in surviving expression and tumor growth	[81,85,86]
Intestine	miRNAs derived from PBEVs have the potential to modulate gut microbiome, intestinal permeability, and mucosal immunity	The results of studies exhibit significant variability due to the lack of consensus regarding PBEVs derived miRNAs	Grapes, grape-fruit, ginger, and carrot	PBEVs, when internalized by macrophages and stem cells, stimulate the HO-1, IL-6, and IL-10 expression, Nrf2 translocation, and activate TCF4 transcription through Wnt activation in the gut tissue	[49,87]
Inflammatory bowel disease, liver disease, cancer	PBEVs have the potential to mediate interspecies communications to exert their anti-oxidant, anti-inflammatory, and regenerative activities	The quantities of proteins derived from PBEVs are lower, and their types differ when compared to MSC-derived EXOs	Blueberry, coconut, ginger, grape-fruit, Hami melon, kiwifruit, orange, pea, pear, soybean, and tomato Blueberry Shiitake mushroom	PBEVs contain miRNAs that can directly interact with mammalian mRNAs of inflammatory mediators. PBEVs inhibit oxidative stress by reducing ROS, Bcl-2, and HO-1, and accelerating Nrf2 translocation, thus ameliorating insulin resistance and liver dysfunction PBEVs mitigate NLRP3 activation by reducing the Casp1 p10 level, and by inhibiting the IL-1β protein assembly through the significant reduction in pro-IL-1β, leading to liver protection	[84,88,89]
Colitis	PBEVs can transport both exogenous drugs and endogenous cargo to epithelial and bacterial cells for their stability in intestinal fluid.	Standardization on mass producing and purification techniques	Ginger Mulberry bark	PBEVs notably decreased the TNF-α, IL-8, and IL-1β mRNA levels by suppressing NF-κB in intestinal cells through their regulatory miRNAs. PBEVs enhanced the AhR-mediated pathway, which is facilitated by HSP70. This leads to COPS8 induction and inhibition of bacterial mRNAs in gut tissue, thus reducing the level of IL-6 and IL-1β.	[90,91,92]
Unspecified	The stability of PBEVs in the digestive system suggests its capability as a functional food to alleviate inflammation.	Instability during isolation and processing with unclear proteomic profiling	Ginger Rice	The uptake of PBEVs by the gut microbiome is governed by its lipid composition, while its RNAs affect bacterial metabolisms and alter the pro-inflammatory cytokines profile. PDENs and their miRNA constituents enhance cell proliferation and GLUT1 expression to regulate blood glucose and metabolism.	[93,94,95]

**Table 3:** Stability studies concerning EVs including PBEVs and animal cell line-based EVs.

Source	Encapsulated API/Bioactive Compound	Stability Conditions	Conclusion	Ref.
PBEVs				
Ginger	Doxorubicin	Stored for 25 days at 4 °C	The experiments showed that Ginger-derived Nano vectors (GDNVs) were still stable and detectable up to 48 h after intravenous injection. This longer residence time provides a longer time window for GDNVs to accumulate at the tumor site.	[59]
Ginger	Shogaols	Obtained negative zeta potential value ranging from 76.2 to 33.5 mV	The magnitude of the measured zeta potential was used to predict the long-term stability of the product.	[156]
Grape-fruit	Exogenous proteins: Exogenous Alexa Fluor 647 labeled bovine serum albumin (BSA) and heat shock protein 70 (HSP70)	The resulting pellet was gently resuspended in 500 μL of PBS with continuous shaking for at least 1 h at 4 °C. The final grapefruit-derived nanovesicle samples were then aliquoted, flash-frozen in liquid nitrogen, and stored at −80 °C until further analysis.	Native PBEVs might be safe and effective carriers of exogenous proteins in human cells	[108]
Grape-fruit	Methotrexate	37 °C for 30 min	Grapefruit-derived nanovesicles were very stable at physiologic temperature (37 °C)	[49]
Grape-fruit	Curcumin	4 °C for more than one month	Grapefruit-derived nano vectors were very stable and did not lose their ability to carry curcumin as well as maintain the biological activity of curcumin.	[157]
Animal-derived EVs				
HEK 293 cell	Penicillin-Streptomycin in medium	Short-term storage conditions: 4 to 90 °C for 30 min Long-term storage conditions: From −70 °C to room temperature (RT) for ten days	EXOs incubated at 60 °C for 30 min showed a slight decrease in HSP70, while 90 °C caused complete protein degradation, indicating that temperatures above 37 °C compromise EXO stability. Markers remained intact at −20 °C and −70 °C, whereas CD63 was lost at 4 °C and RT, with HSP70 partially reduced at RT. EXOs stored at RT showed greater protein and RNA loss compared to those at colder temperatures. Thus, freezing below −20 °C is ideal for long-term storage, and CD63 and HSP70 are more heat-sensitive than CD9.	[158]
Macrophages	Paclitaxel	4 °C, RT, and 37 °C over a period of one month	The remarkable stability of EXOs in aqueous solution was demonstrated over a one-month period at three different temperatures: 4 °C, room temperature (RT), and 37 °C.	[159]
Mouse bronchoalveolar lavage fluid (BALF)		+4 °C and −80 °C	The diameter of BALF-EXOs increased by approximately 10% when stored at +4 °C and by 25% at −80 °C, likely due to the formation of multilamellar structures. These findings suggest that storage conditions can compromise the morphological integrity, surface characteristics, and protein composition of BALF EXOs.	[160]
J774A.1 cells	Curcumin	Short-term storage conditions: Stored in PBS at 37 °C for 3 h Long-term storage conditions: 7 days in PBS at 37 °C	Only 5% of the naked curcumin remained stable. Free curcumin degraded completely within a single day, while only about 8% of albumin-bound or EV-encapsulated curcumin remained stable by day 7. In contrast, 45% of the curcumin in Curcumin Albumin-EVs was stable by day 7. These results highlight that embedding curcumin within CA-EVs significantly enhances its stability, outperforming both albumin-curcumin and EV-loaded curcumin formulations.	[161]
Milk	Paclitaxel	pH 5.0 for 2 h pH 5.8 for 4 h	Incubation in FeSSGF (pH 5.0) for 2 h had no effect on EXO and ExoPAC size, while a slight size increase was observed after 4 h in FeSSIF (pH 5.8).	[1]
EL-4	Curcumin	37 °C for 150 min	Free curcumin degraded rapidly in PBS, with only 25% remaining after 150 min, whereas exosomal curcumin retained over 80% under the same conditions at pH 7.4.	[78]
